# Temporal Trends in Cervical Human Papillomavirus Prevalence Among Females in Xiamen, China (2016-2023): Cross-Sectional Study

**DOI:** 10.2196/70507

**Published:** 2025-10-16

**Authors:** Zhuju Chen, Erjuan Lin, Mo Chen, Shihan Wang, Qiuyuan Lin, Qingquan Chen, Heng Xue, Guanbin Zhang

**Affiliations:** 1 Center of Clinical Laboratory, Zhongshan Hospital Xiamen University, School of Medicine, Xiamen University, Xiamen, China; 2 Department of Laboratory Medicine, Medical Technology Experimental Teaching Center, School of Medical Technology and Engineering, Fujian Medical University, 1 North Xuefu Road, Daxue New District, Fuzhou, Fujian, 350122, China, 86 13811332083, 86 059183569250; 3 Key Laboratory of Clinical Laboratory Technology for Precision Medicine (Fujian Medical University), Fujian Province University, Fuzhou, China; 4 Grade 2023, Stomatology, School and Hospital of Stomatology, Fujian Medical University, Fuzhou, China; 5 Department of Laboratory Medicine, Fujian Maternity and Child Health Hospital College of Clinical Medicine for Obstetrics & Gynecology and Pediatrics, Fujian Medical University, Fuzhou, China; 6 School of Intelligent Medicine, Chengdu University of Traditional Chinese Medicine, Chengdu, China

**Keywords:** human papillomavirus, prevalence, cervical cancer, vaccine, COVID-19 pandemic

## Abstract

**Background:**

Human papillomavirus (HPV) is a primary causative agent of cervical cancer, accounting for more than 90% of cases worldwide. Epidemiological data on regional HPV prevalence and genotype distribution are critical for tailoring targeted cervical cancer prevention strategies, particularly in regions with limited population-based studies.

**Objective:**

This study aimed to investigate temporal trends in the prevalence of overall HPV infection and vaccine-targeted HPV genotypes among females in Xiamen between 2016 and 2023 using annual cross-sectional analyses.

**Methods:**

We analyzed retrospective deidentified data from 63,553 females who underwent HPV genotyping of cervical exfoliated cells at Zhongshan Hospital affiliated with Xiamen University from 2016 to 2023. Data on HPV genotyping, age, and detection time were collected from the hospital’s electronic information system. For each year, we conducted a cross-sectional assessment of HPV infection status to calculate annual HPV prevalence. Temporal trends of HPV prevalence were analyzed across 3 pandemic periods (prepandemic: 2016‐2019, pandemic: 2020‐2022, and postpandemic: 2023) and by age groups.

**Results:**

The overall HPV prevalence was 25.24% (16,039/63,553), comprising high-risk human papillomavirus (HR-HPV) at 19.26% (12,242/63,553) and low-risk human papillomavirus (LR-HPV) at 10.08% (6409/63,553). Vaccine-targeted HPV prevalence rates were bivalent human papillomavirus at 3.56% (2264/63,553), quadrivalent human papillomavirus at 5.89% (3746/63,553), and nine-valent human papillomavirus at 13.64% (8666/63,553), respectively. Notably, the number of non–vaccine-targeted HPV genotypes accounted for 16.01% (10,177/63,553) of all tested females and 63.45% (10,177/16,039) of HPV-positive cases. The top 5 HR-HPV genotypes were HPV52 (3000/63,553, 4.72%), HPV58 (1895/63,553, 2.98%), HPV53 (1582/63,553, 2.49%), HPV16 (1461/63,553, 2.30%), and HPV39 (1116/63,553, 1.76%), while HPV81 (1407/63,553, 2.21%), HPV61 (1268/63,553, 2%), and HPV6 (1101/63,553, 1.73%) were the most prevalent LR-HPV genotypes. Temporal analysis revealed significant declines in the prevalence of overall HPV, HR-HPV, LR-HPV, bivalent human papillomavirus, quadrivalent human papillomavirus, nine-valent human papillomavirus, and specific genotypes (HPV52, HPV58, HPV16, HPV39, and HPV6) from 2016 to 2019 to 2023 (all *P*<.001). Conversely, HPV81 prevalence increased significantly in 2023 compared to 2020‐2022 (2.44% vs 1.96%; *P*<.001). Age-stratified analysis of HPV prevalence showed a significant declining trend with increasing age (*P*<.001), with peak prevalence observed in the ≤20-year age group.

**Conclusions:**

Cervical HPV infection, particularly non–vaccine-targeted genotypes, remains a substantial public health burden in Xiamen, highlighting the urgency to develop broader spectrum vaccines, to enhance cervical cancer screening programs, and to implement age-specific interventions, specifically for females aged ≤20 years. Long-term surveillance of emerging HPV genotypes and vaccination coverage is recommended.

## Introduction

Cervical cancer is the fourth most common cancer and the fourth leading cause of cancer-related death among women, and there were approximately 604,000 cases and 342,000 cancer-related deaths in 2020 globally [1]. In China, it was reported that there were 111,820 new cases and 61,579 deaths of cervical cancer in 2022 [2]. The estimated number of deaths from cervical cancer could even reach 133,000 by 2035 [3]. Therefore, the burden of cervical cancer is still relatively high in China.

Persistent infection with oncogenic human papillomavirus (HPV) genotypes is one of the main causes of cervical cancer [4]. HPV is a circular nonenveloped double-stranded DNA virus, containing approximately 8000 bases. There are more than 200 HPV genotypes that have been identified [5,6]. Conventionally, high-risk human papillomavirus (HR-HPV) includes 12 genotypes classified as Group 1 carcinogens (definitely carcinogenic to humans: HPV16, 18, 31, 33, 35, 39, 45, 51, 52, 56, 58, and 59) and 1 genotype classified as Group 2A (probably carcinogenic to humans: HPV68) [7]. In addition, HPV26, 53, 66, 67, 70, 73, and 82 were classified as agents with limited evidence in humans [8]. A recent study found that there was a causal relationship between invasive cervical cancer and 17 HPV genotypes (HPV16, 18, 45, 33, 31, 58, 52, 59, 26, 69, 35, 39, 73, 68, 56, 82, and 51) [9]. The HPV prevalence of patients with invasive cervical cancer was 48.3 to 1.4 times higher than that of the normal cervical cytology examination population [9], which indicates that the potential carcinogenicity for cervical cancer varied greatly among different HPV genotypes. Therefore, in this study, we analyzed an expanded panel of 17 HR-HPV genotypes, including the 13 conventionally defined HR-HPV genotypes plus 4 additional genotypes (HPV26, 53, 66, and 82) that were frequently included in clinical genotyping assays in our region.

HPV vaccination, as well as cervical cancer screening and treatment, is the main measure to control cervical cancer. Currently, licensed prophylactic HPV vaccines include bivalent vaccines, which target HPV16 and 18 (2V-HPV), quadrivalent vaccines, which target HPV6, 11, 16, and 18 (4V-HPV), and nine-valent vaccines, which cover HPV6, 11, 16, 18, 31, 33, 45, 52, and 58 (9V-HPV). A significant reduction in the prevalence of HPV vaccine genotype and incidence of other HPV-associated diseases had been found in countries with high HPV vaccination [10-12]. However, the HPV vaccine mainly prevents vaccine genotype infection, and the preventive effect on genotypes not covered by the vaccine is quite limited. Therefore, it is necessary to monitor the prevalence and distribution of the HPV genotype dynamically for a long time to provide baseline information for estimating the effectiveness of HPV vaccination, implementing appropriate HPV vaccination strategies, and developing new HPV vaccines.

The prevalence and genotype distribution of HPV exhibited significant differences among countries [13,14], and even varied among different areas within a country [15-19]. In addition, the trend of HPV prevalence also varied among different areas within a province [17,18]. Within the same area, the prevalence and distribution of HPV genotypes also varied in different periods [20]. The prevalence of HPV was also affected by age and the spread of other viruses, with higher prevalence in younger women (<25 years old) [15,21] and older women (≥60 years old) [22].

The COVID-19 epidemic broke out at the end of 2019. To quickly control the epidemic, stringent restrictions on human activities, such as physical distancing and cessation of nonessential activities, were implemented by many countries. The restriction may have an impact on cervical cancer screening, HPV vaccination, and HPV transmission. Recent studies showed that the pandemic had an impact on the prevalence of HPV [21,23]. However, to date, data on HPV prevalence and the distribution of vaccine-targeted genotypes remain limited in Xiamen, particularly across the pre-, intra-, and post–COVID-19 pandemic periods. A global goal of eliminating cervical cancer by 2030 has been set by the World Health Organization. In order to provide more accurate HPV prevalence information, for improving cervical cancer prevention and HPV screening strategies, a comprehensive analysis of HPV prevalence characteristics was conducted in Xiamen from 2016 to 2023.

## Methods

### Study Setting and Data Collection

This study used a cross-sectional design to estimate cervical HPV prevalence and its temporal changes among females at Zhongshan Hospital affiliated with Xiamen University, a tertiary-care academic medical center located in Xiamen, China, from January 2016 to December 2023. Each calendar year (2016‐2023) served as a discrete “time point” for cross-sectional data collection. Annual cross-sectional data were aggregated to evaluate temporal changes in HPV prevalence. The study period was stratified into 3 phases based on the COVID-19 pandemic timeline: prepandemic (2016‐2019), pandemic (2020‐2022), and postpandemic (2023).

Data on HPV genotyping, age, and detection time—originating from the hospital’s health examination centers, outpatient departments, and inpatient wards—were extracted from its electronic information system. To avoid duplicate counting and minimize bias, only data from participants’ first HPV test were included in the analysis, as some individuals underwent multiple tests during the study period. Thus, each year’s dataset represented an independent sample of females tested in that year, with no overlap in the study population across years.

This study adhered to the Strengthening the Reporting of Observational Studies in Epidemiology (STROBE) Statement guidelines for reporting observational studies (STROBE Checklist). A total of 63,553 females who underwent HPV genotyping of cervical exfoliated cells were included in this study. Analyses were stratified by data source and age to minimize bias in HPV prevalence.

The inclusion criteria were as follows: (1) specimens were cervical exfoliated cells, and (2) the period of sample collection was from January 2016 to December 2023. The exclusion criteria were females with incomplete information, such as HPV genotyping results, age, specimen type, and detection time. No age restrictions were applied, and participants were included regardless of their clinical presentation, history of cervical pathology follow-up, pregnancy status, or immunological condition.

### Human Papillomavirus Genotyping

Cervical exfoliated cells were collected by a gynecologist using a cytobrush and stored in a preservation solution for HPV detection. HPV genotyping was performed with the HPV genotyping detection kit (flow-through fluorescence hybridization, Tellgen). The kit integrates multiplex polymerase chain reaction and flow cytometry–based fluorescence hybridization to simultaneously detect 27 HPV genotypes, including 17 HR-HPV (16, 18, 26, 31, 33, 35, 39, 45, 51, 52, 53, 56, 58, 59, 66, 68, and 82), and 10 low-risk human papillomavirus (LR-HPV; 6, 11, 40, 42, 43, 44, 55, 61, 81, and 83) genotypes. Total DNA was extracted from cervical cells using the nucleic acid lysis reagent provided in the kit. This kit used the human β-globin as an internal control to monitor sampling adequacy and amplification efficiency. The assay was conducted on a Luminex200 analyzer (Luminex), which quantified fluorescence intensity for each microsphere. Each genotype was interrogated 100 times, and mean values were calculated to determine positivity. All the experimental procedures strictly followed the manufacturer’s instructions.

### Statistical Analysis

Statistical analyses were performed using SPSS v18.0 (SPSS Inc.), GraphPad Prism v5.0 (GraphPad Software), and Python v3.9.7 (Python Software Foundation). Descriptive statistics for categorical variables were expressed as frequencies and percentages, and continuous variables were presented as median (IQR) values. Annual HPV prevalence was calculated as ([number of individuals with positive HPV test results in a given year]/[total number of eligible individuals tested in that year]×100). The Mann-Kendall test was applied to assess temporal trends in HPV prevalence across the continuous years (2016‐2023). The Cochran-Armitage trend test was used to assess temporal trends in HPV prevalence across 3 periods (2016‐2019, 2020‐2022, and 2023) and by age group. The chi-square test was used to evaluate differences in HPV prevalence between groups. A 2-sided *P* value less than .05 was considered statistically significant.

### Ethical Considerations

The study was conducted following the Declaration of Helsinki principles. Ethical approval was obtained from the Ethics Committee of Zhongshan Hospital affiliated with Xiamen University (approval number 2024‐062). This research constituted a retrospective analysis of previously test data, and all data have been anonymized. In compliance with ethical guidelines, we applied for and were granted a waiver of informed consent, thereby eliminating the need to obtain additional consent from the participants involved. All study data were anonymized and subsequently stored and analyzed within a secure, password-protected database. Information about individuals was removed from all records, and the data were aggregated to prevent the reidentification of any individual. No identifiable information was included in the study or supplementary materials. This study only involved analyzing deidentified data and did not impose any extra financial or other burdens on the participants. Consequently, no compensation, financial or otherwise, was provided to participants included in the study. Furthermore, the study and supplementary files did not contain any images of participants or identifiable materials. Therefore, there was no risk of individual identification in this study.

## Results

### Overall Human Papillomavirus Genotype Distribution (2016-2023)

A total of 63,553 females undergoing cervical exfoliated cell HPV genotyping at Zhongshan Hospital affiliated with Xiamen University from January 2016 to December 2023 were included. Between 2016 and 2023, the average prevalence rates of overall HPV, HR-HPV, LR-HPV, 2V-HPV, 4V-HPV, and 9V-HPV were 25.24% (16,039/63,553), 19.26% (12,242/63,553), 10.08% (6409/63,553), 3.56% (2264/63,553), 5.89% (3746/63,553), and 13.64% (8666/63,553), respectively (Table S1 in Multimedia Appendix 1). Notably, the prevalence of non–vaccine-targeted HPV genotypes was 16.01% (10,177/63,553), accounting for 63.45% (10,177/16,039) of the total positive cases.

**Figure 1.**
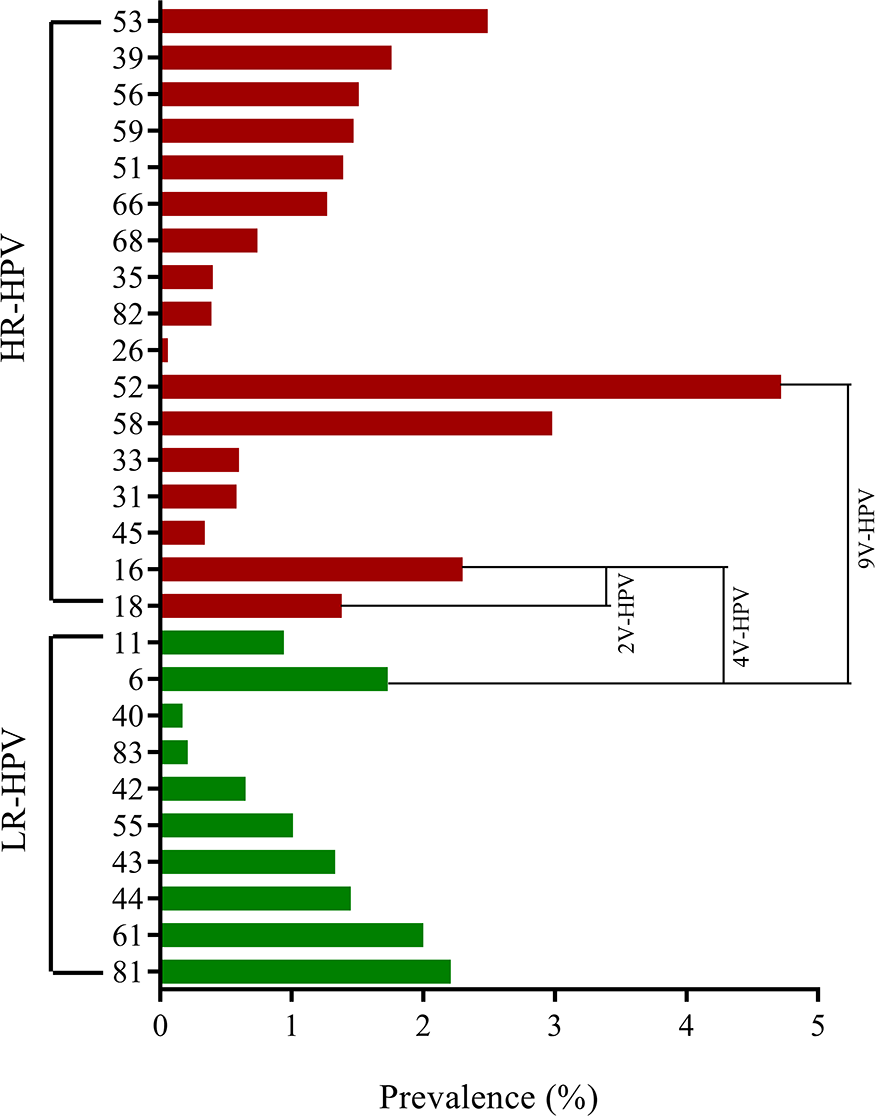
The prevalence of specific human papillomavirus genotypes among females from 2016 to 2023. HR-HPV, LR-HPV, 2V-HPV genotypes (HPV16 and 18) targeted by the bivalent vaccine, 4V-HPV genotypes (HPV16, 18, 6, and 11) targeted by the quadrivalent vaccine, and 9V-HPV genotypes (HPV16, 18, 6, 11, 31, 33, 45, 52, and 58) targeted by the nine-valent vaccine. 2V-HPV: bivalent human papillomavirus; 4V-HPV: quadrivalent human papillomavirus; 9V-HPV: nine-valent human papillomavirus; HPV: human papillomavirus; HR-HPV: high-risk human papillomavirus; LR-HPV: low-risk human papillomavirus.

The prevalence of specific HPV genotypes among females from 2016 to 2023 is shown in Figure 1. The 5 most common HR-HPV genotypes were HPV52 (3000/63,553, 4.72%), HPV58 (1895/63,553, 2.98%), HPV53 (1582/63,553, 2.49%), HPV16 (1461/63,553, 2.30%), and HPV39 (1116/63,553, 1.76%). The top 3 LR-HPV genotypes were HPV81 (1407/63,553, 2.21%), HPV61 (1268/63,553, 2%), and HPV6 (1101/63,553, 1.73%). In particular, among the positive cases, HR-HPV infections accounted for 76.79% (12,317/16,039), while LR-HPV positive cases represented 39.37% (6314/16,039) of the total infections.

### Temporal Trends in the Prevalence of Overall HPV, Vaccine-Targeted HPV, and Specific HPV Genotype (2016-2023)

**Figure 2.**
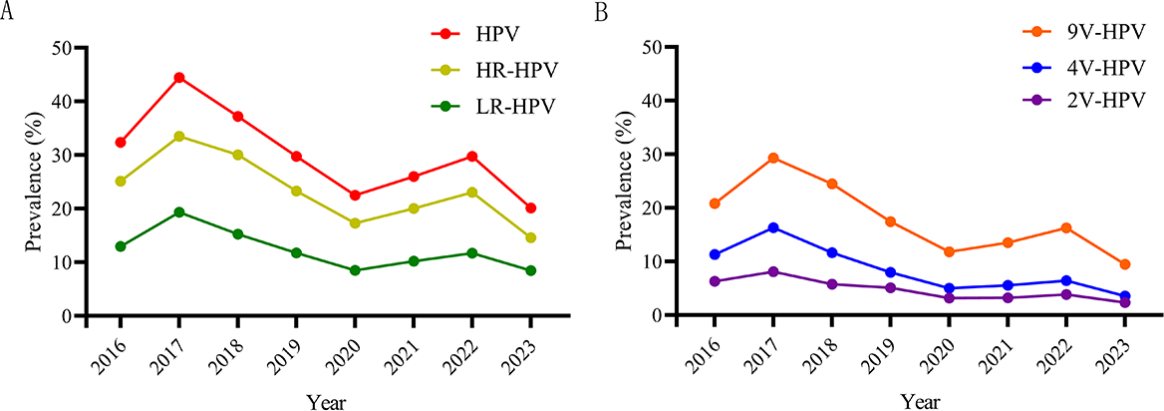
Annual cross-sectional estimates of HPV prevalence among females from 2016 to 2023. (**A**) Overall HPV prevalence, including the prevalence of both HR-HPV and LR-HPV genotypes. (**B**) Prevalence of vaccine-targeted HPV genotypes. 2V-HPV genotypes (HPV16 and 18) targeted by the bivalent vaccine, 4V-HPV genotypes (HPV16, 18, 6, and 11) targeted by the quadrivalent vaccine, and 9V-HPV genotypes (HPV16, 18, 6, 11, 31, 33, 45, 52, and 58) targeted by the nine-valent vaccine. The Mann-Kendall test was applied to assess temporal trends in HPV prevalence across 2016‐2023. 2V-HPV: bivalent human papillomavirus; 4V-HPV: quadrivalent human papillomavirus; 9V-HPV: nine-valent human papillomavirus; HPV: human papillomavirus; HR-HPV: high-risk human papillomavirus; LR-HPV: low-risk human papillomavirus.

The annual prevalence trends of overall HPV, HR-HPV, and LR-HPV demonstrated a declining trajectory (overall HPV: *P*=.03; HR-HPV: *P*=.04; LR-HPV: *P*=.02), generally exhibiting a distinct “M-shaped” pattern. This pattern featured 2 peaks in 2017 and 2022, along with 3 troughs in 2016, 2020, and 2023. In 2023, the prevalence rates of overall HPV, HR-HPV, and LR-HPV were 20.15% (3994/19,826), 14.61% (2897/19,826), and 8.46% (1678/19,826), respectively (Figure 2A and Table S1 in ).

All vaccine-targeted HPV genotypes (2V-HPV, 4V-HPV, and 9V-HPV) exhibited significant declining trends from 2016 to 2023 (2V-HPV: *P*=.04; 4V-HPV: *P*=.02; 9V-HPV: *P*=.01) (Figure 2B; Table S1 in ). The prevalences of 9V-HPV and 4V-HPV showed greater fluctuations, whereas 2V-HPV prevalence remained relatively stable. Notably, the first prevalence peak in 2017 occurred shortly after the first HPV vaccine received regulatory approval in China in 2016, potentially reflecting increased HPV testing following the formal clinical introduction of vaccines. The subsequent rise from the 2020 trough to the 2022 peak overlapped with the COVID-19 pandemic.

**Figure 3.**
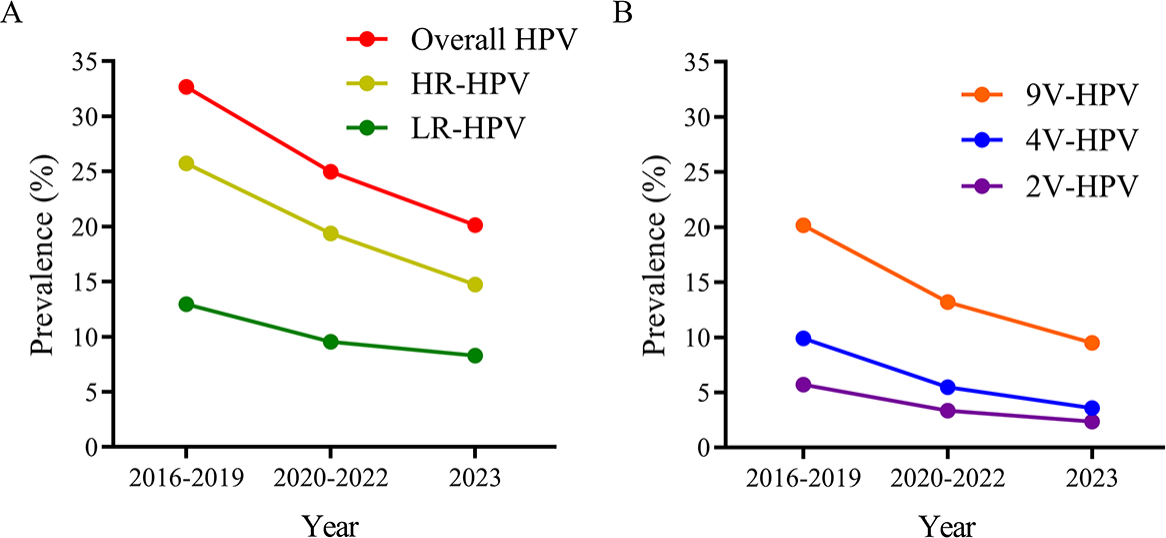
HPV prevalence among females stratified by time periods: 2016‐2019, 2020‐2022, and 2023. (**A**) Overall HPV prevalence, including the prevalence of both HR-HPV and LR-HPV genotypes. (**B**) Prevalence of vaccine-targeted HPV genotypes. 2V-HPV genotypes (HPV16 and 18) targeted by the bivalent vaccine, 4V-HPV genotypes (HPV16, 18, 6, and 11) targeted by the quadrivalent vaccine, and 9V-HPV genotypes (HPV16, 18, 6, 11, 31, 33, 45, 52, and 58) targeted by the nine-valent vaccine. The Cochran-Armitage trend test was applied to assess the differences in HPV prevalence among the 2016‐2019, 2020‐2022, and 2023 groups. 2V-HPV: bivalent human papillomavirus; 4V-HPV: quadrivalent human papillomavirus; 9V-HPV:nine-valent human papillomavirus; HPV: human papillomavirus; HR-HPV: high-risk human papillomavirus; LR-HPV: low-risk human papillomavirus.

Stratifying by time periods (2016‐2019, 2020‐2022, and 2023) revealed consistent downward trends (all *P*<.001) in overall and vaccine-targeted HPV prevalence (Figure 3A and B; Table S2 in ). Compared to 2016‐2019, overall HPV prevalence in 2023 decreased by 38.34% (32.68% vs 20.15%), while HR-HPV and LR-HPV prevalence decreased by 42.89% (25.58% vs 14.61%) and 35.81% (13.18% vs 8.46%), respectively. For vaccine-targeted genotypes, 2V-HPV prevalence decreased by 58.88% (5.69% vs 2.34%), 4V-HPV by 63.84% (9.90% vs 3.58%), and 9V-HPV by 52.97% (20.18% vs 9.49%) over the same period. These findings confirm significant reductions in HPV burden during and after the COVID-19 pandemic.

**Figure 4.**
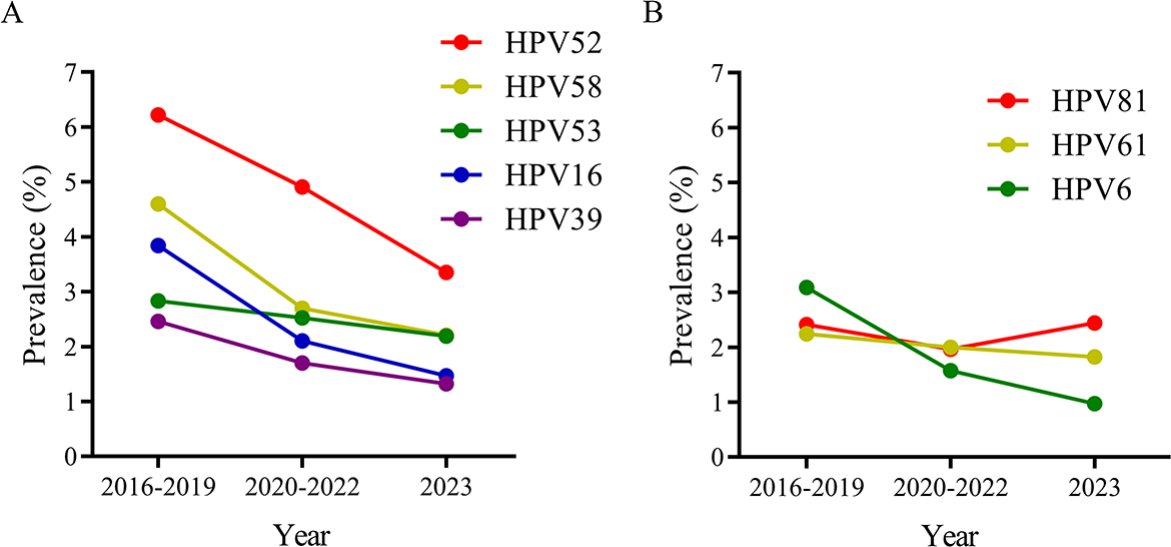
Prevalence of the top 5 high-risk HPV and top 3 low-risk HPV genotypes among females, stratified by time periods: 2016‐2019, 2020‐2022, and 2023. (**A**) The top 5 high-risk HPV genotypes. (**B**) The top 3 low-risk HPV genotypes. The Cochran-Armitage trend test was applied to assess the differences in HPV prevalence among the 2016‐2019, 2020‐2022, and 2023 groups. The chi-square test also revealed a significant difference in HPV81 prevalence between the 2023 and 2020‐2022 groups (2.44% vs 1.96%; *P*<.001). HPV: human papillomavirus.

We further examined how the most prevalent HR-HPV and LR-HPV genotypes changed across the 3 time periods (2016‐2019, 2020‐2022, and 2023) to align with broader trends (Figure 4A and B; Table S3 in ). All 5 top HR-HPV genotypes showed significant downward trends (all *P*<.001): HPV52 (46.14% reduction), HPV58 (51.96% reduction), HPV53 (22.61% reduction), HPV16 (61.72% reduction), and HPV39 (46.34% reduction) from 2016‐2019 to 2023. Notably, HPV39, which is not targeted by current vaccines, also exhibited a marked decline. Among the top LR-HPV genotypes, HPV61 and HPV6 also showed significant decreases from 2016‐2019 to 2023 (18.75% reduction for HPV61; *P*=.003; and 68.61% reduction for HPV6; *P*<.001). In contrast, HPV81 displayed a V-shaped trend with no overall significant temporal pattern (*P*=.46); however, its prevalence increased from 1.96% in 2020‐2022 to 2.44% in 2023 (*P*<.001).

Collectively, these findings highlight dynamic shifts in HPV epidemiology over the 8-year period, with widespread declines in the prevalence of overall HPV, HR-HPV, LR-HPV, and vaccine-targeted HPV genotypes, alongside distinct trends in specific genotypes.

### Prevalence of Overall HPV and Vaccine-Targeted Genotypes by 5-Year Age Intervals (2016–2023)

**Figure 5.**
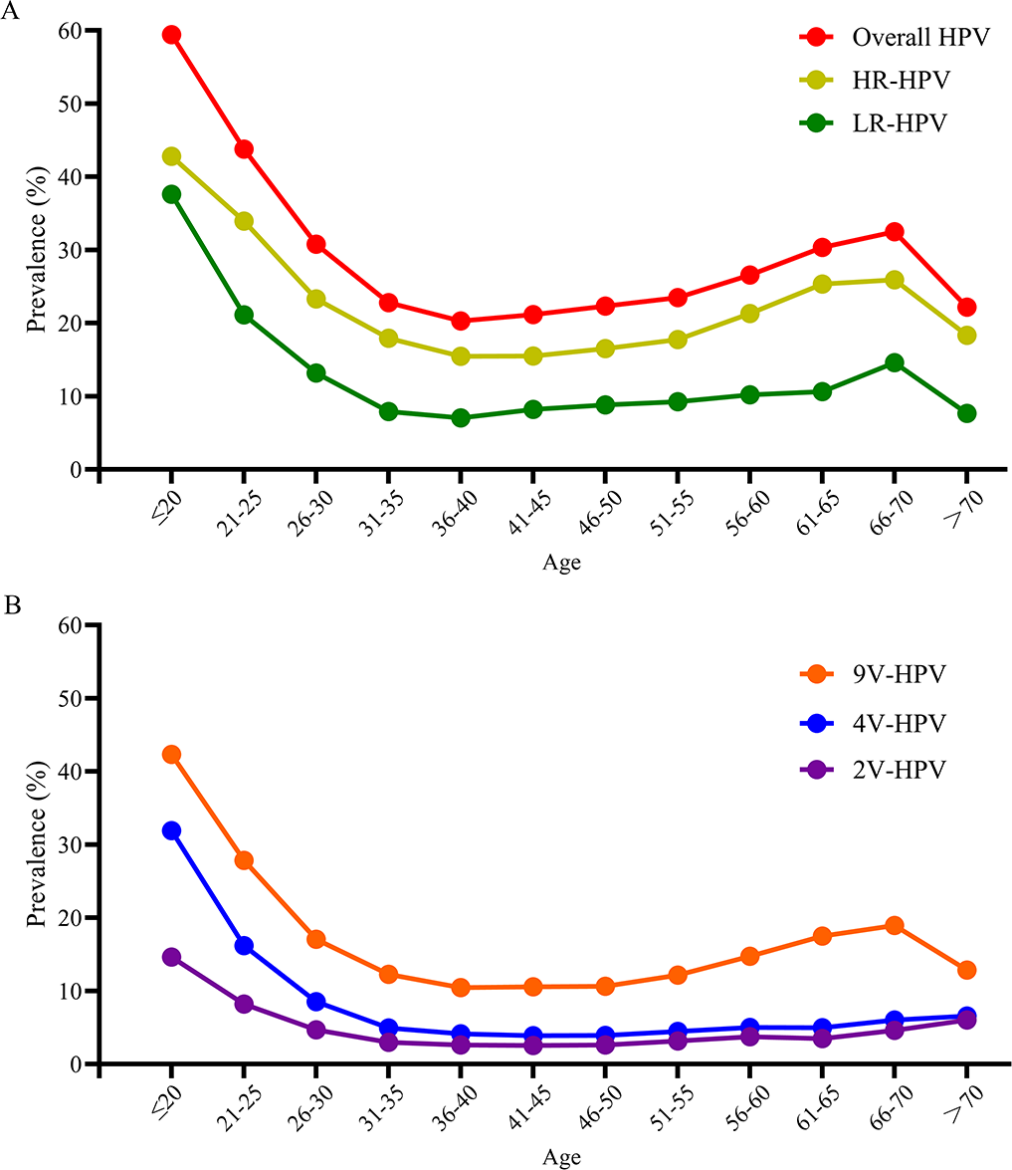
Prevalence of overall HPV and vaccine-targeted HPV genotypes by 5-year age intervals (2016‐2023). (**A**) Overall HPV prevalence, including the prevalence of both HR-HPV and LR-HPV genotypes. (**B**) Prevalence of vaccine-targeted HPV genotypes. 2V-HPV genotypes (HPV16 and 18) targeted by the bivalent vaccine, 4V-HPV genotypes (HPV16, 18, 6, and 11) targeted by the quadrivalent vaccine, and 9V-HPV genotypes (HPV16, 18, 6, 11, 31, 33, 45, 52, and 58) targeted by the nine-valent vaccine. The Cochran-Armitage trend test was used to assess the trend of HPV prevalence changes across age groups. 2V-HPV: bivalent human papillomavirus; 4V-HPV: quadrivalent human papillomavirus; 9V-HPV: nine-valent human papillomavirus; HPV: human papillomavirus; HR-HPV: high-risk human papillomavirus; LR-HPV: low-risk human papillomavirus.

To investigate the influence of age on HPV infection, we assessed the prevalence of overall HPV and vaccine-targeted HPV genotypes among females stratified by 5-year age intervals. Overall, the prevalence of HPV (including overall HPV, HR-HPV, LR-HPV, 2V-HPV, 4V-HPV, and 9V-HPV) showed a significant declining trend with increasing age (*P*<.001). Specifically, HPV prevalence varied significantly by age, with distinct patterns observed across 5-year intervals (Figure 5A and B; Table S4 in ). The prevalence of overall HPV, HR-HPV, LR-HPV, and 9V-HPV peaked in the ≤20 years age group, with prevalence of 59.45% (365/614) for overall HPV, 42.83% (263/614) for HR-HPV, 37.62% (231/614) for LR-HPV, and 42.35% (260/614) for 9V-HPV, respectively, followed by a gradual decline in the 21-25 and 26- to 30-year age groups. Prevalence remained relatively stable from 31‐35 to 51‐55 years, then increased slowly starting at 56‐60 years, with a smaller secondary peak observed at 66‐70 years before declining again in the >70-year group. For 2V-HPV and 4V-HPV, prevalence also peaked in the ≤20-year age group at 14.66% (90/614) for 2V-HPV and 31.92% (196/614) for 4V-HPV, respectively, followed by a gradual decline in the 21‐ to 25-year age group and stable prevalence thereafter (26‐30 to >70 years). Stratification by 5-year intervals revealed that the youngest age group (≤20 years) consistently exhibited the highest prevalence of both overall HPV and vaccine-targeted genotypes, underscoring the importance of early screening and vaccination initiatives in this population.

## Discussion

### Principal Findings

The infection of HPV in the cervical area, especially the persistent infection of HR-HPV, is closely related to cervical cancer. The high prevalence of HPV poses a challenge to the prevention and control of cervical cancer. HPV prevalence is an important basis for analyzing and formulating cervical cancer prevention and control strategies. The prevalence and genotype distribution of HPV varied in different areas, even in the same areas during different periods. Our results showed that the prevalence of overall HPV was 25.24% (16,039/63,553), which aligned with findings from Zhejiang (22.3% in all participants and 27.3% in the gynecological clinic subgroup) [24]. However, our HPV prevalence was higher than the 18.4% reported in Xiamen [17] and the 8.60% observed in Putian, Fujian Province [18]. Several factors may account for the observed differences. First, the number of HPV genotypes included in the testing differed. Our study analyzed 27 HPV genotypes, whereas the Xiamen [17] and Putian [18] studies only included 21 genotypes. Second, population composition significantly influences HPV prevalence. Inpatient and outpatient populations often include individuals with cervical cancer or precancerous lesions, who tend to have higher HPV prevalence. In contrast, health examination populations primarily consist of asymptomatic individuals undergoing routine screening, resulting in lower HPV prevalence. Our study included participants from health examination centers, outpatient departments, and inpatient wards. Consequently, the overall HPV prevalence was similar to that reported in the study by Yan et al [24] for a similar population group and fell between the rates observed in health examination and outpatient groups. Third, temporal variations in HPV prevalence may influence prevalence differences. Our study spanned 2016‐2017, during which the HPV prevalence was relatively high compared to that reported by Lin et al [18]. Consistent with Yao et al [17], our data also showed a downward trend in the prevalence of overall HPV, HR-HPV, and LR-HPV from 2016 to 2023. Our findings indicate that the HPV prevalence among females in Xiamen is still relatively high, although showing a downward trend over the years.

Several studies have identified the top 5 HR-HPV genotypes as HPV52, 16, 58, 51, and 39 in Beijing [25], Luoyang of Henan Province [26], Xiamen [17], and Putian [18] of Fujian Province. Notably, our study and a recent investigation in Chengdu-Chongqing (2014‐2023) [27] consistently observed HPV52, 58, 53, 16, and 39 as the predominant genotypes, with HPV53 emerging as a novel top-ranked genotype across regions. HPV53 is not targeted by authorized licensed HPV vaccines currently. Even the classification of HPV53 is still unclear. The International Agency for Research on Cancer has not yet classified it as HR-HPV, while an increasing number of studies [16,18,19,26-28] have done so. Therefore, it is essential to enhance HPV53 detection and research on its association with cervical cancer.

While LR-HPV genotypes such as HPV6 and HPV11 are mainly associated with genital warts [29,30] with low malignant potential, persistent infections impose significant burdens on the patient’s appearance, psychology, and finances [27,31]. HPV6 and HPV11 also have long been considered the most common LR-HPV genotypes. However, recent studies have found that HPV81 is the most common LR-HPV genotype in exfoliated cervical cells [19,27], a finding corroborated by our study. Notably, HPV81 exhibited a rising prevalence trend (1.96% in 2020‐2022 vs 2.44% in 2023), contrasting with the declining prevalence of HPV6. This upward trajectory aligns with a recent study showing HPV81 as the most frequently detected LR-HPV genotype in exfoliated cervical cells, with a statistically significant increasing trend [27]. Given that HPV81 exhibits strong survival competitiveness and is not included in any licensed HPV vaccines, its prevalence trend and pathogenicity require urgent attention.

HPV vaccination is a primary strategy for preventing HPV infection and associated diseases. A meta-analysis involving 60 million individuals showed an 80% reduction in the prevalence of HPV16 and HPV18 more than 8 years postvaccination [32]. Currently, licensed prophylactic HPV vaccines mainly include the bivalent, quadrivalent, and nine-valent vaccines. Our research shows that the prevalence rates of 2V-HPV, 4V-HPV, and 9V-HPV among females in Xiamen were 3.56% (2264/63,553), 5.89% (3746/63,553), and 13.64% (8666/63,553), respectively, with a downward trend observed from 2016 to 2023. While vaccine coverage expansion likely contributed to declining vaccine-genotype HPV prevalence, this trend cannot be exclusively attributed to immunization programs. Confounding factors, including shifts in screening practices, sexual behavior dynamics, and demographic changes over the study period, may also influence these outcomes. Notably, the relatively high persistence of 9V-HPV prevalence underscores the continued importance of expanding vaccination coverage as a key component of comprehensive strategies to prevent and control HPV infection and its associated sequelae, including cervical cancer.

HPV prevalence exhibited an age-specific pattern, with a significant age-related decline observed across age groups. In our study, the highest prevalence was observed among females aged ≤20 years, aligning with findings from previous studies [22,26]. This elevated prevalence in younger females may be attributed to frequent sexual activity and lack of immunity to HPV [33]. Thus, targeted interventions are warranted, including the development of age-tailored vaccination strategies and prioritization of HPV immunization for females aged ≤20 years.

As a sexually transmitted virus, HPV prevalence is influenced not only by sexual behavior patterns and activity levels [34] but also by a complex interplay of behavioral, socioeconomic, and environmental factors. Human activity has been profoundly changed due to the emergence of the COVID-19 pandemic. Several studies found that the COVID-19 pandemic harmed cancer screening and vaccination [35,36]. Our analysis identified a notable decline in the prevalence of overall HPV, HR-HPV, LR-HPV, 2V-HPV, 4V-HPV, and 9V-HPV; and specific genotypes, such as HPV52, HPV58, HPV16, HPV39, and HPV6 during and postpandemic compared to prepandemic periods—a finding corroborated by other regional studies [19,21]. Our study found that HPV testing volumes peaked at 19,826 in 2023 and 14,886 in 2020 during the study period, likely driven by policy interventions: a free domestic bivalent HPV vaccination program launched in September 2020 and free cervical cancer screening initiatives introduced in October 2022. These policies probably motivated greater participation in HPV testing, including asymptomatic or healthy women. The COVID-19 pandemic also influenced testing uptake indirectly. Thus, the observed decline in HPV prevalence reflects synergistic effects of pandemic-related behavioral changes, heightened health awareness, and expanded access to preventive services.

### Limitations

This study has several limitations. First, detailed information regarding HPV vaccination for each subject was not obtained, and an in-depth investigation into the influence of economic and cultural factors on HPV prevalence was lacking. Second, selection bias may exist as hospital-based data primarily reflect testing behaviors rather than the true prevalence at the population level. Third, we did not document participants’ symptom status, cervical conditions, reproductive status, or immunological status. Consequently, it was impossible to differentiate between participants seeking care due to symptoms or undergoing follow-up for cervical conditions and those undergoing routine screening. This prevented us from exploring the relationship between HPV prevalence and the incidence rates of cervical cancer or cervical epithelial cell lesions. Pregnant women and immunocompromised individuals may exhibit distinct HPV infection patterns. We could neither exclude nor characterize these subgroups due to incomplete clinical documentation. This may introduce heterogeneity into the study population, as HPV prevalence and clearance rates could be influenced by hormonal changes during pregnancy or immune dysfunction. Fourth, detailed data on residential location, sociodemographic factors, behaviors, and clinical covariates were unavailable, hindering stratified analyses of their impact on HPV prevalence. These limitations underscore the necessity for future studies to proactively collect comprehensive data on clinical, geographic, demographic, and behavioral aspects to enable stratified analyses of vulnerable subgroups.

### Conclusions

HPV prevalence among females in Xiamen remains high, declining from 2016 to 2023. HPV52, HPV58, HPV53, HPV16, and HPV39 were the top 5 HR-HPV genotypes in Xiamen, whereas HPV81 was the most prevalent LR-HPV genotype, with an upward trend after the COVID-19 pandemic. Non–vaccine-targeted HPV genotypes demonstrated high prevalence, highlighting the need for developing and implementing broader spectrum HPV vaccines. In addition, further attention should be paid to the prevention and control of HPV infection in females aged ≤20 years.

## Supplementary material

10.2196/70507 Multimedia Appendix 1 Data on human papillomavirus prevalence.

10.2196/70507 Checklist 1 Strengthening the Reporting of Observational Studies in EpidemiologyReporting of Observational Studies in Epidemiology statement and data on human papillomavirus prevalence.

## Abbreviations

2V-HPV: bivalent human papillomavirus
4V-HPV: quadrivalent human papillomavirus
9V-HPV: nine-valent human papillomavirus
HPV: human papillomavirus
HR-HPV: high-risk human papillomavirus
LR-HPV: low-risk human papillomavirus
STROBE: Strengthening the Reporting of Observational Studies in Epidemiology
