# Supplementary material for: Temporal Trends in Cervical Human Papillomavirus Prevalence Among Females in Xiamen, China (2016-2023): Cross-Sectional Study
Source: JMIR Public Health Surveill. 2025 Oct 16;11:e70507. doi: 10.2196/70507 (PMC12530691; doi:10.2196/70507)
Supplement: Multimedia Appendix 1 [file publichealth-v11-e70507-s001.doc]

**Table S1.** Annual cross-sectional estimates of HPV prevalence among females from 2016 to 2023 in Xiamen, China.

| Year | Total (n) | Age Median (IQR) | Overall HPV | | HR-HPV | | LR-HPV | | 2V-HPV | | 4V-HPV | | 9V-HPV | |
| --- | --- | --- | --- | --- | --- | --- | --- | --- | --- | --- | --- | --- | --- | --- |
|  |  |  | Positive (n) | Prevalence (%) | Positive (n) | Prevalence  (%) | Positive (n) | Prevalence  (%) | Positive (n) | Prevalence  (%) | Positive (n) | Prevalence  (%) | Positive (n) | Prevalence  (%) |
| Total | 63,553 | 40.0  (32.0-48.0) | 16,039 | 25.24 | 12,242 | 19.26 | 6,409 | 10.08 | 2,264 | 3.56 | 3,746 | 5.89 | 8,666 | 13.64 |
| 2016 | 2,786 | 39.0  (31.0-46.0) | 903 | 32.41 | 700 | 25.13 | 361 | 12.96 | 176 | 6.32 | 315 | 11.31 | 580 | 20.82 |
| 2017 | 1,286 | 36.0  (29.0-44.0) | 572 | 44.48 | 431 | 33.51 | 249 | 19.36 | 104 | 8.09 | 210 | 16.33 | 377 | 29.32) |
| 2018 | 2,171 | 37.0  (30.0-46.0) | 808 | 37.22 | 653 | 30.08 | 332 | 15.29 | 125 | 5.76 | 253 | 11.65 | 531 | 24.46 |
| 2019 | 8,297 | 39.0  (31.0-47.0) | 2,469 | 29.76 | 1,935 | 23.32 | 975 | 11.75 | 423 | 5.10 | 661 | 7.97 | 1,446 | 17.43 |
| 2020 | 14,886 | 40.0  (32.0-48.0) | 3,349 | 22.50 | 2,580 | 17.33 | 1,265 | 8.50 | 474 | 3.18 | 750 | 5.04 | 1,756 | 11.80 |
| 2021 | 8,356 | 39.0  (32.0-48.0) | 2,175 | 26.03 | 1,675 | 20.05 | 853 | 10.21 | 268 | 3.21 | 465 | 5.56 | 1,128 | 13.50 |
| 2022 | 5,945 | 39.0  (32.0-49.0) | 1,769 | 29.76 | 1,371 | 23.06 | 696 | 11.71 | 230 | 3.87 | 383 | 6.44 | 967 | 16.27 |
| 2023 | 19,826 | 41.0  (34.0-50.0) | 3,994 | 20.15 | 2,897 | 14.61 | 1,678 | 8.46 | 464 | 2.34 | 709 | 3.58 | 1,881 | 9.49 |
| *P*-value | | | * | | * | | * | | * | | * | | * | |

Overall HPV prevalence，including the prevalence of both HR-HPV and LR-HPV genotypes. 2V-HPV genotypes (HPV16 and 18) targeted by the bivalent vaccine, 4V-HPV genotypes (HPV16, 18, 6, and 11) targeted by the quadrivalent vaccine, and 9V-HPV genotypes (HPV16, 18, 6, 11, 31, 33, 45, 52, and 58) targeted by the nine-valent vaccine. The Mann-Kendall test was applied to assess temporal trends in HPV prevalence across 2016‐2023. 2V-HPV: bivalent human papillomavirus; 4V-HPV: quadrivalent human papillomavirus; 9V-HPV: nine-valent human papillomavirus; HPV: human papillomavirus; HR-HPV: high-risk human papillomavirus; LR-HPV: low-risk human papillomavirus; IQR: interquartile range; *, *P*＜.05.

**Table S2.** HPV prevalence among females stratified by time periods: 2016–2019, 2020–2022, and 2023.

| Group | 2016-2019 (n=14,540) | 2020-2022 (n=29,187) | 2023 (n=19,826) | P Value |
| --- | --- | --- | --- | --- |
| Overall HPV |  |  |  |  |
| Positive (n) | 4,752 | 7,293 | 3,994 | *** |
| Prevalence (%) | 32.68 | 24.99 | 20.15 |
| HR-HPV |  |  |  |  |
| Positive (n) | 3,719 | 5,626 | 2,897 | *** |
| Prevalence (%) | 25.58 | 19.28 | 14.61 |
| LR-HPV |  |  |  |  |
| Positive (n) | 1,917 | 2,814 | 1,678 | *** |
| Prevalence (%) | 13.18 | 9.64 | 8.46 |
| 2V-HPV |  |  |  |  |
| Positive (n) | 828 | 972 | 464 | *** |
| Prevalence (%) | 5.69 | 3.33 | 2.34 |
| 4V-HPV |  |  |  |  |
| Positive (n) | 1,439 | 1,598 | 709 | *** |
| Prevalence (%) | 9.90 | 5.48 | 3.58 |
| 9V-HPV |  |  |  |  |
| Positive (n) | 2,934 | 3,851 | 1,881 | *** |
| Prevalence (%) | 20.18 | 13.19 | 9.49 |

2V-HPV genotypes (HPV16 and 18) targeted by the bivalent vaccine, 4V-HPV genotypes (HPV16, 18, 6, and 11) targeted by the quadrivalent vaccine, and 9V-HPV genotypes (HPV16, 18, 6, 11, 31, 33, 45, 52, and 58) targeted by the nine-valent vaccine. The Cochran-Armitage trend test was applied to assess the differences in HPV prevalence among the 2016-2019, 2020-2022 and 2023 groups. 2V-HPV: bivalent human papillomavirus; 4V-HPV: quadrivalent human papillomavirus; 9V-HPV:nine-valent human papillomavirus; HPV: human papillomavirus; HR-HPV: high-risk human papillomavirus; LR-HPV: low-risk human papillomavirus; ***, *P* ＜ .001.

**Table S3.** Prevalence of the top 5 HR-HPV and top 3 LR-HPV genotypes among females, stratified by time periods: 2016–2019, 2020–2022, and 2023.

| Group | | 2016-2019 (n=14,540) | 2020-2022 (n=29,187) | 2023 (n=19,826) | P Value |
| --- | --- | --- | --- | --- | --- |
| Top 5 HR-HPV | | |  |  |  |
| HPV52 | |  |  |  |  |
| Positive (n) | | 904 | 1,432 | 664 | *** |
| Prevalence (%) | | 6.22 | 4.91 | 3.35 |
| HPV58 | |  |  |  |  |
| Positive (n) | | 669 | 788 | 438 | *** |
| Prevalence (%) | | 4.60 | 2.70 | 2.21 |
| HPV53 | |  |  |  |  |
| Positive (n) | | 411 | 736 | 435 | *** |
| Prevalence (%) | | 2.83 | 2.52 | 2.19 |
| HPV16 | |  |  |  |  |
| Positive (n) | | 558 | 612 | 291 | *** |
| Prevalence (%) | | 3.84 | 2.10 | 1.47 |
| HPV39 | |  |  |  |  |
| Positive (n) | | 358 | 496 | 262 | *** |
| Prevalence (%) | | 2.46 | 1.70 | 1.32 |
| Top 3 LR-HPV | | |  |  |  |
| HPV81 |  | |  |  |  |
| Positive (n) | 351 | | 573 | 483 | ns |
| Prevalence (%) | 2.41 | | 1.96 | 2.44 |
| HPV61 |  | |  |  |  |
| Positive (n) | 325 | | 583 | 360 | ** |
| Prevalence (%) | 2.24 | | 2.00 | 1.82 |
| HPV6 |  | |  |  |  |
| Positive (n) | 449 | | 459 | 193 | *** |
| Prevalence (%) | 3.09 | | 1.57 | 0.97 |

The Cochran-Armitage trend test was applied to assess the differences in HPV prevalence among the 2016-2019, 2020-2022 and 2023 groups. The Chi-square test also revealed a significant difference in HPV81 prevalence between the 2020–2022 and 2023 groups (*P*<.001). HPV: human papillomavirus; HR-HPV: high-risk human papillomavirus; LR-HPV: low-risk human papillomavirus; **, *P*＜.01; ***, *P*＜.001; ns, none significance difference.

**Table S4.** Prevalence of overall HPV and vaccine-targeted HPV genotypes by 5-year age intervals (2016–2023).

| Age | Total (n) | Overall HPV | | HR-HPV | | LR-HPV | | 2V-HPV | | 4V-HPV | | 9V-HPV | |
| --- | --- | --- | --- | --- | --- | --- | --- | --- | --- | --- | --- | --- | --- |
|  |  | Positive (n) | Prevalence  (%) | Positive (n) | Prevalence  (%) | Positive (n) | Prevalence  (%) | Positive (n) | Prevalence  (%) | Positive (n) | Prevalence  (%) | Positive (n) | Prevalence  (%) |
| ≤20 | 614 | 365 | 59.45 | 263 | 42.83 | 231 | 37.62 | 90 | 14.66 | 196 | 31.92 | 260 | 42.35 |
| 21-25 | 3,936 | 1,724 | 43.80 | 1,336 | 33.94 | 832 | 21.14 | 323 | 8.21 | 638 | 16.21 | 1,096 | 27.85 |
| 26-30 | 7,616 | 2,346 | 30.80 | 1,777 | 23.33 | 1,006 | 13.21 | 358 | 4.70 | 652 | 8.56 | 1,300 | 17.07 |
| 31-35 | 10,537 | 2,405 | 22.82 | 1,892 | 17.96 | 837 | 7.94 | 316 | 3.00 | 523 | 4.96 | 1,296 | 12.30 |
| 36-40 | 10,290 | 2,091 | 20.32 | 1,592 | 15.47 | 726 | 7.06 | 272 | 2.64 | 428 | 4.16 | 1,076 | 10.46 |
| 41-45 | 9,490 | 2,009 | 21.17 | 1,472 | 15.51 | 781 | 8.23 | 242 | 2.55 | 372 | 3.92 | 1,005 | 10.59 |
| 46-50 | 9,312 | 2,079 | 22.33 | 1,539 | 16.53 | 822 | 8.83 | 246 | 2.64 | 367 | 3.94 | 992 | 10.65 |
| 51-55 | 6,061 | 1,424 | 23.49 | 1,076 | 17.75 | 562 | 9.27 | 192 | 3.17 | 271 | 4.47 | 737 | 12.16 |
| 56-60 | 3,172 | 843 | 26.58 | 676 | 21.31 | 324 | 10.21 | 119 | 3.75 | 159 | 5.01 | 468 | 14.75 |
| 61-65 | 1,400 | 425 | 30.36 | 355 | 25.36 | 149 | 10.64 | 49 | 3.50 | 70 | 5.00 | 245 | 17.50 |
| 66-70 | 760 | 247 | 32.50 | 197 | 25.92 | 111 | 14.61 | 35 | 4.61 | 46 | 6.05 | 144 | 18.95 |
| >70 | 365 | 81 | 22.19 | 67 | 18.36 | 28 | 7.67 | 22 | 6.03 | 24 | 6.58 | 47 | 12.88 |
| *P*-value | | ******* | | ******* | | ******* | | ******* | | ******* | | ******* | |

Overall HPV prevalence，including the prevalence of both HR-HPV and LR-HPV genotypes. 2V-HPV genotypes (HPV16 and 18) targeted by the bivalent vaccine, 4V-HPV genotypes (HPV16, 18, 6, and 11) targeted by the quadrivalent vaccine, and 9V-HPV genotypes (HPV16, 18, 6, 11, 31, 33, 45, 52, and 58) targeted by the nine-valent vaccine. The Cochran-Armitage trend test was used to assess the trend of HPV prevalence changes across age groups. 2V-HPV: bivalent human papillomavirus; 4V-HPV: quadrivalent human papillomavirus; 9V-HPV: nine-valent human papillomavirus; HPV: human papillomavirus; HR-HPV: high-risk human papillomavirus; LR-HPV: low-risk human papillomavirus; ***, *P*＜.001.
